# CEPI Workshop Report: Applying Disease X Vaccine Library and Knowledge Base Approaches to Severe Fever with Thrombocytopenia Syndrome (SFTS)

**DOI:** 10.3390/vaccines14040304

**Published:** 2026-03-28

**Authors:** Mitsutaka Kitano, Byoung-Shik Shim, Hitoshi Sasaki, Jonathan F. Lovell, V. Narry Kim, Rachel Kim, Wei-Chao Huang, Sun Bean Kim, Woo-Jung Park, Alison A. Bettis, Keun Hwa Lee, Yuki Takamatsu, Javier Castillo-Olivares, Rokusuke Yoshikawa, Jimmy D. Gollihar, Thomas H. Segall-Shapiro, Keith C. Spencer, Gene Malin, Nora M. Gerhards, Polina Brangel, Lindi Dalland, Soo-Young Kwon, Satoshi Kaneko, Kouichi Morita, Manki Song, Timothy Endy

**Affiliations:** 1 Coalition for Epidemic Preparedness Innovations, Askekroken 11, 0277 Oslo, Norway; 2 International Vaccine Institute, Seoul 08826, Republic of Korea; 3 Institute of Tropical Medicine, Nagasaki University, Nagasaki City 852-8523, Japan; 4 POP Biotechnologies, Inc., 1576 Sweet Home Rd., Buffalo, NY 14228, USA; 5 School of Biological Sciences, Seoul National University, Seoul 08826, Republic of Korea; 6 ST Pharm Co., Ltd., Seoul 06170, Republic of Korea; 7 Center for Vaccine Research, National Institute of Health, Cheongju 28160, Republic of Korea; 8 Hanyang University College of Medicine, Seoul 04763, Republic of Korea; 9 National Research Center for the Control and Prevention of Infectious Diseases, Nagasaki University, Nagasaki City 852-8523, Japan; 10 Center for Infectious Diseases, Houston Methodist Research Institute, Houston, TX 77030, USA; 11 Wageningen University and Research, Wageningen Bioveterinary Research, 8221 RA Lelystad, The Netherlands

**Keywords:** Severe Fever with Thrombocytopenia Syndrome, SFTS, SFTSV, *Dabie bandavirus*, vaccine

## Abstract

On 9–10 December 2025, the Coalition for Epidemic Preparedness Innovations (CEPI) and the International Vaccine Institute (IVI) convened a workshop in Seoul under CEPI’s Disease X Program. The primary objective was to identify existing gaps needing to be filled and streamline vaccine development and preparedness for Severe Fever with Thrombocytopenia Syndrome (SFTS). CEPI’s partners and experts discussed a multifaceted agenda, ranging from understanding the evolving epidemiology to the refinement of animal models and immunological assay harmonization. Key outcomes included the refinement of Target Product Profiles (TPPs) specifying use cases for both peacetime and outbreak contexts, alongside a recommendation for a core immunoassay panel aimed at harmonizing evaluation frameworks and mitigating the challenges posed by low SFTS prevalence. Integration of the One Health approach emerged as a critical strategy for SFTS prevention, complemented by proactive regulatory engagement to compress vaccine development timelines. This report summarizes these key insights from the workshop, delineating a strategic framework for delivering safe, effective, and accessible vaccines for SFTS and broader Disease X threats.

## 1. Introduction

Severe Fever with Thrombocytopenia Syndrome (SFTS), caused by *Dabie bandavirus* (family *Phenuiviridae*, order *Bunyavirales*), is a tick-borne viral hemorrhagic fever with a high fatality rate for those with symptomatic disease [1,2]. Despite being recognized as a priority pathogen by several Asian countries and international public health organizations, including the World Health Organization (WHO), no licensed human vaccine is currently available [3]. This gap underscores the need for accelerated and coordinated development efforts. Such efforts align with the “Disease X” concept, which was formally added to the WHO R&D Blueprint in March 2017 to address pathogens capable of causing serious epidemics, including both unknown agents and known pathogens with increased transmissibility or severity [3].

The Coalition for Epidemic Preparedness Innovations (CEPI), established in 2017, aims to accelerate the development of vaccines and countermeasures for epidemic and pandemic threats, including potentially emerging pathogens [4]. To achieve this, CEPI’s Disease X Vaccine Library Program is an integrated approach driven by CEPI’s 100 Days Mission to have a vaccine available for the next Disease X emergence. The program involves developing an extensive knowledge base for each virus family, applying artificial intelligence (AI)-driven computational approaches to vaccine design, and evaluating these designs across a variety of vaccine platforms through preclinical and clinical development [5]. The *Phenuiviridae* family includes a number of emerging pathogens including Rift Valley fever, Toscana, and Heartland viruses [6]. Data generated through SFTS vaccine development, such as antigen design, animal model performance, and human safety profiles, will be integrated into the Disease X Knowledge Base to refine its predictive capabilities. Simultaneously, the SFTS vaccine candidate contributes to the Vaccine Library as an exemplar that can be adapted for related viruses with close phylogenetic ties to SFTS.

CEPI currently supports three SFTS vaccine development projects, each utilizing distinct and innovative platform technologies (Table 1).

The Nagasaki University project employs an advanced self-assembling mRNA delivery system, Nanoball, which offers freeze-drying capability and high physicochemical stability, facilitating streamlined manufacturing and flexible dosage forms. This project integrates AI-driven antigen design in collaboration with NEC OncoImmunity [7]. The POP Biotechnologies project leverages SNAP™ (Spontaneous Nanoliposome Antigen Particle) technology, a protein-based platform that displays antigens on immunogenic lipid nanoparticles. This “plug-and-play” technology was validated through the development of a COVID-19 vaccine candidate evaluated in Phase 3 trials [8,9]. Finally, the International Vaccine Institute (IVI) leads a consortium integrating proprietary SmartCap^®^ technology from ST Pharm with LNP-formulated mRNA, validated during Phase 1 trials, utilizing structure-based modeling and AI-driven antigen design in collaboration with the Korea Disease Control Agency (KDCA) and Seoul National University (SNU) [10,11]. Importantly, the IVI is establishing WHO-standard SFTS reference materials for antibody assays to ensure harmonized evaluation [12]. While data from these SFTS projects will strengthen the Disease X Knowledge Base and serve as exemplar vaccines, there is an increasing need to advance these candidates toward formal licensure for use in endemic regions. Despite the initiation of all of these important initiatives, substantial scientific and technical gaps remain [13]. These challenges must be addressed to translate these innovative vaccine platforms into accessible public medical countermeasures (MCMs).

To address these gaps, in December 2025, CEPI and IVI convened a multidisciplinary group of researchers to refine the strategies underpinning the Disease X Vaccine Library and knowledge base approach and to discuss streamlining SFTS vaccine development. This group comprised experts in preclinical and clinical research, epidemiology, and global health from diverse sectors, including academic institutions (Nagasaki University, Seoul National University, and Hanyang University), industry partners (POP Biotechnologies and ST Pharm), and research institutes (the Korea National Institute of Health, Wageningen University and Research, and the Houston Methodist Research Institute). The meeting focused on identifying critical gaps in research, epidemiology, Target Product Profile (TPP) development, and evaluation method harmonization necessary to advance SFTS vaccine development. The primary objectives were: (1) to share recent SFTS research findings and prioritize remaining scientific and technical gaps; (2) to explore multifaceted TPPs for SFTS vaccines suitable for peacetime or outbreak contexts, including harmonized evaluation methods to streamline vaccine development; and (3) to facilitate collaboration and knowledge exchange among global experts. This report summarizes the key insights generated from these presentations and discussions.

## 2. Surveillance Systems and Epidemiological Gaps

SFTS is primarily endemic in China, South Korea, and Japan, with recent evidence indicating a steady expansion of its endemic range within these countries [14,15,16]. The SFTS virus (SFTSV) has also emerged in other Asian nations, including Vietnam in 2017; Thailand, Taiwan, and Myanmar in 2019; and Pakistan in 2020 [17,18,19,20,21]. Although the primary transmission vector, *Haemaphysalis longicornis*, is widely distributed across Asia, North America, and Australia [22,23], confirmed human cases remain documented only within Asia to date. Nevertheless, the prevalence of related pathogens, such as Heartland virus in the United States, underscores a significant risk for the future emergence of SFTS or similar phenuiviral diseases beyond current endemic zones [24].

Workshop presentations highlighted a global upward trend in reported SFTS cases, as corroborated by recent systematic reviews [25]. Interestingly, while the annual incidence is rising, the Case Fatality Rate (CFR) appears to be declining or stabilizing in some regions. In South Korea, the initial CFR of 47% in 2013 has decreased and stabilized at the mid-teens, a figure that remains higher than those reported in China and Japan [26]. In Japan, surveillance data from the Japan Institute for Health Security (JIHS) revealed 1242 cumulative cases and 126 deaths since 2013, with a notable geographic expansion from western regions toward northern Japan, including Hokkaido [27]. Although the reported CFR stands at 10.6% in Japan, participants cautioned that this may be an underestimate, as current surveillance often reflects only initial diagnostic data. While the apparent decline in CFR may reflect improvements in diagnostic accuracy, enhancements in epidemiological surveillance systems, and improved clinical interventions, the surge in case numbers in previously non-endemic districts suggests that SFTS is gaining significant epidemiological momentum. The primary gaps identified include the need for internationally harmonized surveillance protocols and standardized case definitions, as well as more granular data on subclinical infections, all of which are critical for accurately determining disease burden and defining clinical trial endpoints for vaccine evaluation.

Presenters emphasized that demographic and occupational data indicate that advanced age is the primary risk factor for severe disease, increased complications, and high mortality. In South Korea and Japan, agricultural workers and rural populations over 60 remain the most vulnerable due to both their age and their frequent exposure to tick-infested environments through agricultural activities [15,28,29]. Beyond human exposure, the workshop highlighted the critical role of the animal–human interface in disease transmission. In Japan, feline cases have exhibited a CFR exceeding 60%, suggesting that outdoor-roaming companion animals may drive domestic transmission risks [30]. Similarly, Taiwan’s nationwide surveillance in ruminants and wildlife has revealed high viral RNA detection rates, underscoring the importance of zoonotic monitoring in understanding transmission dynamics [19]. Temporal patterns in China, South Korea, and Japan show that incidence consistently peaks during mid-summer, aligning with heightened tick activity and human outdoor activity [28,31]. Notably, the expansion of the monthly average incidence window suggests a growing public health burden during these peak periods. Presenters underscored critical gaps in surveillance across the countries. While endemic countries maintain mandatory reporting systems integrated with tick monitoring, nations with sporadic cases often rely on reactive, outbreak-linked investigations rather than continuous surveillance [31,32]. This discrepancy limits the understanding of the true disease burden and highlights the urgent need for harmonized global reporting structures and predictive modeling to identify emerging transmission hotspots.

To refine the “force of infection” and streamline vaccine development, participants identified several urgent research priorities that span epidemiological, clinical, and biological areas. These efforts require improved epidemiological characterization, particularly in clarifying the role of subclinical infections and refining demographic profiles through stratified attack rates [33,34]. Furthermore, investigating transmission mechanisms remains essential, with a focus on vector competence across different tick species and the specific pathways of human-to-human and zoonotic transmission including which amplifying host species dominate in different regions and their relative contribution to human spillover [1,35,36]. These efforts must be complemented by an exploration of viral and immunological dynamics, including the study of genomic diversity across regions and its association with clinical severity, alongside the long-term persistence of both natural and vaccine-derived immunity [37,38,39]. Presenters emphasized that resolving these scientific knowledge gaps demands a robust One Health approach, integrating virology, veterinary medicine, and entomology, to establish standardized modeling and evidence-based vaccine strategies [40,41]. Within this One Health framework, a critical research gap remains regarding entomological control measures. Specifically, more focus is needed on strategies to suppress tick populations and disrupt their breeding habitats, which would provide a fundamental barrier not only for SFTS but also for other co-endemic tick-borne diseases.

These epidemiological and surveillance gaps directly impact the feasibility and design of future clinical trials. Without harmonized case definitions and granular incidence data from diverse regions, determining precise sample sizes for clinical trials remains a significant challenge. Furthermore, the lack of standardized diagnostics hinders the ability to identify suitable trial sites with high “force of infection,” potentially leading to prolonged timelines or compromised study feasibility. Resolving these methodological and operational hurdles is therefore not only a public health priority but a technical prerequisite for establishing validated clinical endpoints acceptable to regulatory bodies. Ultimately, such a comprehensive approach is imperative given the high CFR, pandemic potential, and the increasing international concern across multiple stakeholders.

## 3. Challenges in Defining Target Product Profiles (TPPs) for SFTS Vaccines

The workshop emphasized the urgent need to establish a robust TPP for SFTS vaccines, a critical framework currently absent for this emerging zoonotic threat. A TPP serves as a strategic target for developers and regulatory bodies, translating clinical needs into measurable product attributes and directly informing the design of preclinical studies by specifying performance criteria [42]. Participants stressed the importance of developing comprehensive objectives that encompass three distinct clinical scenarios:

- Use Case A: Reactive Deployment During Outbreaks
  Prioritizes a rapid onset of protective immunity, ideally within 7 to 14 days following the initial dose. This requirement influences dosing strategies, potentially necessitating a single-dose regimen to ensure timely intervention in an outbreak context. While a two-dose schedule remains a benchmark for evaluating the anamnestic response required for durable protection, the urgency of reactive use may prioritize immediate seroconversion.
- Use Case B: Prophylaxis for High-Risk Populations in Endemic Regions
  Prioritizes long-term immunity, with protection targets ranging from one to three years. This durability, combined with efficacy targets, underscores the necessity of extending longitudinal preclinical studies to validate long-term performance under this scenario. Furthermore, given the epidemiological significance of advanced age in SFTS severity, these TPPs must specifically address the need for high immunogenicity and safety in older populations [43,44].
- Use Case C: Veterinary/One Health Applications Targeting Animal Reservoirs
  Aims to support the development of vaccines for both human and veterinary use to achieve comprehensive disease control [45]. This strategy requires addressing the distinct regulatory and economic requirements of veterinary vaccines, including significantly lower Cost of Goods sold (CoGs) thresholds and different safety profiles [46]. This includes defining modifications to animal study protocols to support additional indications, ensuring the TPP’s relevance across both human and animal health domains.

Additionally, presenters highlighted persistent challenges in defining the precise target population. While SFTS disproportionately affects older adults who experience higher morbidity and mortality, significant gaps remain regarding exposure rates and subclinical disease burden among younger cohorts [47]. To address these uncertainties and potentially disrupt transmission chains, participants suggested that the TPP must remain flexible enough to accommodate vaccination strategies beyond the elderly, particularly for occupationally exposed younger individuals and key animal reservoirs such as livestock and companion animals. TPP development must also integrate logistical and strategic considerations. Decision making regarding product format, balancing the public health advantages of multi-dose containers for mass campaigns against the commercial viability and ease of use of single-dose formats, is essential [48]. Furthermore, parameters such as shelf life, cold-chain requirements, and CoGs are pivotal for ensuring deployment feasibility, especially in the context of the CEPI’s 100 Days Mission.

## 4. Harmonization of Immunological Assays and Endpoints

Workshop participants emphasized that harmonizing analytical methods is critical for the success of SFTS vaccine development, particularly given the low disease prevalence that constrains large-scale Phase 3 efficacy trials. In the absence of large-scale clinical data, establishing robust Correlates of Protection (CoPs) through standardized evaluations becomes essential for regulatory approval [49]. Transitioning from project-specific metrics to harmonized standards will maximize the collective value of research findings, allowing for more robust benchmarking across different vaccine candidates.

Discussions highlighted the importance of aligning core immunological assays across laboratories. Participants stressed that the calibration of neutralization assays using an international standard reference serum is critical [50,51,52]. This ensures that neutralization titers are expressed in standardized units, enabling the direct comparison of vaccine potency and immunogenicity across diverse platforms. Furthermore, presenters underscored that antibody levels measured by both ELISA and the virus neutralizing assay were designated as primary immunogenicity endpoints. The development of a validated pseudovirus neutralization assay as a proxy for live virus neutralization could be beneficial [53]. The role of T-cell immunity remains a subject of intense deliberation. Workshop attendees noted challenges in interpreting T-cell data, such as results from ELISpot or intracellular cytokine staining, and underscored the need to validate these assays as potential secondary endpoints. Currently, a significant knowledge gap remains regarding the relative contributions of humoral versus cellular immunity to clinical protection [54]. Consequently, while T-cell immunity is currently classified as a desirable research objective in this workshop, it may become a mandatory requirement as more evidence emerges regarding its necessity for durable or cross-reactive protection. Attendees noted that achieving complete analytical comparability requires addressing the lack of harmonization in secondary parameters, including standardized virus titration methods and the use of consistent cell lines for assays.

Workshop presentations and group discussions highlighted the critical role of standardized animal challenge models in evaluating vaccine efficacy. Preclinical evaluation across projects will primarily utilize the type-I interferon receptor (IFNAR) knockout mouse model, using protection against clinical signs and lethality, quantification of viral load in internal organs, and assessment of pathological lesions as the primary readouts [55,56,57]. Beyond the mouse model, participants highlighted the value of the aged ferret model. Unlike young ferrets, aged ferrets more closely replicate the severe clinical manifestations and high mortality observed in older human populations, providing a clinically relevant assessment of vaccine-induced protection in high-risk groups [58,59,60,61]. However, given the logistical constraints in securing aged ferrets, the workshop strongly recommended refining the young ferret model, such as through viral dose titration or specific strain selection, to improve its sensitivity as a surrogate for severe SFTS studies [62]. To maximize data consistency, the selection of a globally representative challenge virus genotype was discussed. A major concern remains the breadth of cross-protection across diverse SFTSV genotypes, as recent genomic analyses reveal active evolutionary dynamics through multiple reassortment genotypes even while the virus maintains a relatively conservative genetic landscape with nucleotide sequence identity exceeding 93% across its entire genome [63]. While SFTSV is currently considered a single serotype, suggesting that a single candidate could logically confer pan-genotypic immunity, this must be empirically confirmed through panel neutralization assays using representative genotypes [57,58]. Furthermore, even with a single serotype, continuous genetic surveillance is vital to monitor potential future shifts in the virus’s antigenicity, pathogenicity, and transmissibility to ensure long-term vaccine effectiveness and determine if periodic strain updates will be required in the future. Additionally, the current variability in efficacy endpoints, including survival, viral load, and pathology, underscores the necessity of harmonizing a core metric indicator. Presenters emphasized that the harmonization is intended not to rank different technologies, but to enable a transparent comparison of the unique characteristics and immunological profiles across different vaccine platforms.

Finally, participants emphasized the critical importance of establishing a unified CoP. Attendees agreed that the primary clinical objective should focus on preventing severe disease and mortality. The discussion highlighted the utility of passive serum transfer studies not only to define protective antibody thresholds but also to confirm the sufficiency of humoral immunity alone [54]. A key remaining gap is whether the CoP will rely solely on neutralizing antibody titers or necessitate a combined humoral and cellular immune response for robust protection. Furthermore, participants noted that the specific CoP may vary depending on the TPP scenario. For instance, a CoP focused on rapid, short-term protection for reactive use may differ from the metrics required to validate long-term, durable immunity in prophylactic contexts.

## 5. Favipiravir Case Study: An Illustrative Regulatory Precedent for Rare, High-Fatality Indications Where RCTs Are Impractical

Although the workshop primarily focused on vaccine development, the inclusion of a case study on Favipiravir provided a valuable complementary perspective on regulatory pathways for rare, high-fatality diseases where randomized controlled trials (RCTs) are often impractical. Favipiravir, a broad-spectrum antiviral targeting RNA-dependent RNA polymerase [64,65], demonstrated preclinical and clinical efficacy against SFTSV [66,67,68], providing critical insights for managing emerging viral threats.

Clinical evidence from Japan, derived from Investigator-Initiated Trials (2016–2018), demonstrated reduced mortality and a significant correlation between baseline viral load and survival outcomes [67]. Due to the rarity of the disease, a subsequent Phase 3 study employed the use of external controls, confirming a 50% reduction in mortality compared with historical cohorts [68]. Complementary findings from China corroborated these results, emphasizing that early intervention is a critical determinant of clinical success [69,70]. This lesson underscores the importance of vaccine-induced immunity that can rapidly control viral replication post exposure, preventing the transition to severe clinical stages, which is a key objective for the SFTS vaccine TPP.

Based on this cumulative evidence, Favipiravir received conditional approval in Japan in June 2024. This case was discussed as an illustrative regulatory precedent, demonstrating the pragmatic evaluation required when conventional RCTs are unfeasible. Approval was predicated on an integrated evidence package combining preclinical data, limited human clinical trials, and platform experience. This model of regulatory innovation holds significant relevance for vaccines targeting Disease X, where traditional efficacy data may be constrained.

The case further highlighted the utility of adaptive trial designs and external control methodologies, which were accepted as scientifically justified alternatives. Workshop participants discussed how these actionable strategies could accelerate vaccine development timelines by integrating cross-sectoral insights from various MCMs, including antivirals. Key considerations included establishing evaluation frameworks that leverage diverse evidence and ensuring early coordination among academia, regulatory agencies, and industry [71]. Finally, the Favipiravir experience underscores that managing emerging threats requires a flexible strategy, where the proactive harmonization of global data and standards is essential to facilitate timely access to interventions in response to public health threats.

## 6. Strategic CMC Frameworks for Disease X and Outbreak Responses

Disease X preparedness forms the strategic foundation of CEPI’s 100 Days Mission, and its success depends on embedding Chemistry, Manufacturing, and Controls (CMC) principles throughout the vaccine development lifecycle. To operationalize this strategy, the workshop introduced two complementary resources: the CEPI’s CMC Framework and the CMC Rapid Response Framework [72,73]. Both are designed to accelerate vaccine development, manufacturing, and deployment across diverse outbreak scenarios, providing a roadmap for emerging threats like SFTS.

Workshop presentations emphasized that CMC is not merely a technical requirement but a cornerstone of pandemic preparedness. It encompasses product characterization, manufacturing process optimization, and rigorous quality control systems to ensure consistency and regulatory compliance. Importantly, these frameworks translate multidisciplinary concepts into actionable tools that facilitate accelerated manufacturing timelines and rapid scale-up during a crisis.

The CEPI’s CMC Framework provides stage-specific deliverables to enhance the probability of success from preclinical evaluation through to commercialization [72]. Broadly applicable to platforms such as viral vectors, mRNA, and nanoparticle-based technologies, it can be adapted for any viral family. Structurally, it is organized as a two-dimensional matrix mapping five critical domains: manufacturing process development, formulation/stability, analytical methods, supply chain, and compliance. This systematic tool enables the early identification and mitigation of potential bottlenecks, ensuring that technical readiness remains aligned with clinical development timelines.

In parallel, the CMC Rapid Response Framework operationalizes Disease X preparedness by aligning with CEPI’s 100 Days Mission [73]. It addresses three outbreak scenarios: 1. known pathogens, where existing vaccines and manufacturing platforms enable rapid scale-up; 2. related pathogens, leveraging established templates and platform technologies with moderate adaptation (a category highly relevant to SFTS); 3. novel pathogens, which necessitate de novo development and present the greatest challenge. Projected timelines for vaccine readiness under these scenarios are 100 days, 150 days, and 200–250 days, respectively. These ambitious targets underscore the need for proactive platform validation, regulatory harmonization, and the ready-state deployment of standardized supply chain components. Workshop discussions concluded that expanding Disease X vaccine libraries and associated knowledge bases, supported by robust CMC strategy and data, is vital to mitigating the risks posed by future outbreaks.

## 7. Equitable Access in Disease X Preparedness

Equitable access constitutes a foundational principle of CEPI and remains central to accelerating vaccine development for both recognized threats and Disease X [74]. From the perspective of Disease X preparedness, equitable access requires that vaccines are not only technically viable but also physically available and financially affordable to populations at greatest risk.

Workshop presentations introduced the concept of “Zero-Day Contracting” as a means to eliminate legal bottlenecks impeding rapid response. This model shifts the paradigm from exhaustive bilateral negotiations to a streamlined “click-to-accept” framework for early-stage research. By increasing risk tolerance at the outset, this process can be applied to a broad portfolio of preclinical projects, reserving intensive legal and commercial negotiations for candidates that successfully transition to clinical trials.

Participants also discussed that developing vaccines for limited commercial markets, such as SFTS, necessitates a robust partnership ecosystem. While CEPI provides the “push” through R&D funding, effective “pull” incentives must include commitments to sustainable procurement and manufacturing capacity [4]. To encourage industry participation, partnerships must allow developers to retain intellectual property while embedding tiered-pricing or access provisions within the development pipeline. This ensures that even if a product is licensed for high-income markets, pathways for equitable availability in low- and middle-income countries (LMICs) remain secure.

The workshop further addressed the importance of a networked research environment to foster rapid data and material exchange. By bifurcating outputs into non-proprietary research data and protectable intellectual property, such networks can facilitate open knowledge sharing while safeguarding commercial rights necessary for downstream investment. Within this framework, pre-negotiated, standardized licensing terms, such as reasonable royalties for sequence utilization, can avert the legal encumbrances that hindered progress during previous outbreaks.

Additionally, presenters underscored that transitioning from physical Material Transfer Agreements (MTAs) to Data Transfer Agreements (DTAs) for viral sequences can substantially reduce administrative lead times and navigate biosecurity barriers. By streamlining these processes and advancing the positioning of manufacturing capacity in endemic regions, the global health community can ensure that scientific breakthroughs are translated into tangible public health outcomes with unprecedented speed and equity.

## 8. Conclusions

The workshop underscored that the importance of SFTS vaccine development is both a regional public health necessity and a strategic imperative within the broader CEPI Disease X Programme. As a high-fatality pathogen with expanding geographical reach, development of an SFTS vaccine as an exemplar refines the rapid response frameworks envisioned in CEPI’s 100 Days Mission. Achieving this requires a multifaceted strategy that begins with integrating One Health surveillance and epidemiological characterization to align TPPs with harmonized analytical methods.

To transition from strategic discussion to operational execution, the workshop identified several priority next steps. These include finalizing the SFTS TPPs based on the three defined use-case scenarios and establishing a clear governance and ownership framework for reference standards. Furthermore, participants agreed on the urgent need to formalize a standardized challenge strain panel and assays, while initiating a structured development plan for defining CoP. Establishing clear milestones for early and continuous engagement with international regulatory bodies was also emphasized as a technical prerequisite for success.

By harmonizing evaluation frameworks and defining these technical benchmarks, the global health society can mitigate the challenges of low disease prevalence and provide the scientific rigor needed to de-risk vaccine candidates. These advancements must be supported by equitable access principles, ensuring that legal and administrative barriers are addressed early through streamlined contracting and data-sharing mechanisms.

In conclusion, continued efforts to stimulate knowledge exchange and build solid networks, as facilitated by this workshop, are fundamental to the success of SFTS vaccine development and Disease X prevention and control. By bridging academia, industry, and regulatory bodies through this unified strategic framework, scientific breakthroughs can be translated into tangible public health outcomes. These collective measures are indispensable to enhancing global health and ensuring that the innovations developed for SFTS serve as resilient tools against the next emerging threat.

## Figures and Tables

**Table 1:** Summary of CEPI-funded SFTS Vaccine Platforms.

Lead Institution	Platform Type	Key Advantages	Research Scope
Nagasaki University	mRNA-Nanoball	Nanoballs represent a novel mRNA delivery architecture using nano-sized, negatively charged particles that serve as a robust alternative to traditional lipid nanoparticles (LNPs). By integrating AI-driven rational antigen design with the enhanced structural integrity of the mRNA carrier, the technology offers superior thermal stability, supporting lyophilization and extended storage at either refrigerated or room temperatures.	Preclinical studies
POP Biotechnologies	Protein-SNAP	SNAP^TM^ (Spontaneous Nanoliposome Antigen Particle) technology utilizes a “plug-and-play” architecture to display antigens on cobalt-modified liposomes via an affinity-based coupling method. This allows for rapid assembly and streamlined manufacturing, as safety and immunogenicity profiles were validated in Phase 3 trials of the EuCorVac-19 COVID-19 vaccine.	Preclinical studies
International Vaccine Institute	mRNA-LNP	The mRNA-LNP platform, leveraging SmartCap^®^ technologies, provides safety and immunogenicity profiles validated through Phase 1 clinical trials. This platform integrates advanced AI-driven antigen design with proprietary stabilizing UTR (untranslated region) sequences to maximize mRNA translation efficiency and effectiveness.	Preclinical and Phase I/II clinical trials

## Data Availability

Not applicable.
